# CloVR: A virtual machine for automated and portable sequence analysis from the desktop using cloud computing

**DOI:** 10.1186/1471-2105-12-356

**Published:** 2011-08-30

**Authors:** Samuel V Angiuoli, Malcolm Matalka, Aaron Gussman, Kevin Galens, Mahesh Vangala, David R Riley, Cesar Arze, James R White, Owen White, W Florian Fricke

**Affiliations:** 1 Institute for Genome Sciences (IGS), University of Maryland School of Medicine, Baltimore, Maryland, USA; 2 Center for Bioinformatics and Computational Biology, University of Maryland, College Park, Maryland, USA

## Abstract

**Background:**

Next-generation sequencing technologies have decentralized sequence acquisition, increasing the demand for new bioinformatics tools that are easy to use, portable across multiple platforms, and scalable for high-throughput applications. Cloud computing platforms provide on-demand access to computing infrastructure over the Internet and can be used in combination with custom built virtual machines to distribute pre-packaged with pre-configured software.

**Results:**

We describe the Cloud Virtual Resource, CloVR, a new desktop application for push-button automated sequence analysis that can utilize cloud computing resources. CloVR is implemented as a single portable virtual machine (VM) that provides several automated analysis pipelines for microbial genomics, including 16S, whole genome and metagenome sequence analysis. The CloVR VM runs on a personal computer, utilizes local computer resources and requires minimal installation, addressing key challenges in deploying bioinformatics workflows. In addition CloVR supports use of remote cloud computing resources to improve performance for large-scale sequence processing. In a case study, we demonstrate the use of CloVR to automatically process next-generation sequencing data on multiple cloud computing platforms.

**Conclusion:**

The CloVR VM and associated architecture lowers the barrier of entry for utilizing complex analysis protocols on both local single- and multi-core computers and cloud systems for high throughput data processing.

## Background

The cost of genome sequencing has been rapidly decreasing due to the introduction of a number of new affordable next-generation sequencing technologies. Coupled with the decreasing costs is an increase in the volume of data produced by sequencing machines. As a consequence, the genomics field has been rapidly changing: Larger amounts of sequence data are not only being produced at lower costs, but also more and more often by small to midsize research groups outside of the large sequencing centers all over the world [1]. This is a trend, which is likely to continue, as newer generation sequencing technologies continue to drive down costs.

High-throughput sequencing technologies have decentralized sequence acquisition, increasing the number of research groups in demand of sequence analysis all over the world. The increasing volume of data from next-generation sequencing has led to increased computational and bioinformatics needs and concern of a bioinformatics bottleneck [2]. Technical challenges in use of bioinformatics software [3,4] and difficulties in utilization of available computational resources [5,6] impede analysis, interpretation and full exploration of sequence data.

The installation, operation, and maintenance of software tools for bioinformatics analysis can be cumbersome and require significant technical expertise leading to efforts that pre-package and bundle bioinformatics tools [3]. While, many bioinformatics software tools routinely used in sequence analysis are open source and freely available, the installation, operation, and maintenance can be cumbersome and require significant technical expertise [3,7]. In addition, individual tools are often insufficient for sequence analysis and, rather, need to be integrated with others into multi-step pipelines for thorough analysis. To aid with this, bioinformatics workflows systems and workbenches, such as Galaxy [8], Ergatis [9], GenePattern [10], Taverna [11] provide user interfaces to simplify execution of tools and pipelines on centralized servers. Prior to analysis, researchers utilizing genomics approaches are faced with a multitude of choices of analysis protocols and best practices are often poorly documented [12]. Complexities of analysis pipelines and lack of transparent protocols can limit reproducibility of computed results [4]. Use of workbenches that store pipeline metadata and track data provenance can improve reproducibility [8].

Bioinformatics service providers, such as RAST [13], MG-RAST [14], ISGA [15], and the IGS Annotation engine [16], have attempted to address challenges in microbial genome analysis by providing centralized services, where users submit sequence data to a web site for analysis using standardized pipelines. In this model, the service provider operates the online resource, dedicating the necessary personnel and computational resources to support a community of users. Bioinformatics workflow systems [8-11] also operate on central servers, utilizing dedicated or shared network based storage, and clusters of computers for improved processing throughput. Other efforts have bundled tools into portable software packages for installation on a local computer, including Mothur [17] and Qiime [18] for 16S ribosomal RNA analysis and DIYA [19] for bacterial genome annotation. A virtual machine (VM) encapsulates an operating system with pre-installed and pre-configured software in a single executable file that can be distributed and run elsewhere. VMs provide a means to eliminate complex software installations and adaptations for portable execution, directly addressing one of the challenges involved with using bioinformatics tools and pipelines.

Cloud computing offers leasable computational resources on-demand over a network [20]. The cloud computing model can simplify access to a variety of computing architectures, including large memory machines, while eliminating the need to build or administer a local computer network addressing challenges in access and deployment of infrastructure for bioinformatics [6,21]. Cloud computing platforms have been emerging in the commercial sector, including the Amazon Elastic Compute Cloud (EC2) [22], and in the public sector to support research, such as Magellan [23] and DIAG [24]. In combination with virtual machines, cloud computing can help improve accessibility to complex bioinformatics workflows in a reproducible fashion on a readily accessible distributed computing platform [25].

There is considerable enthusiasm in the bioinformatics community for use of cloud computing in sequence analysis [6,21,25,26]. While cloud computing platforms that provide ready access to computing resources over the Internet on-demand can improve processing throughput, utilization of bioinformatics tools and pipelines on such distributed systems requires technical expertise to achieve robust operation and intended performance gains [6,27]. Cluster management software, workflow systems, or databases may be installed, maintained, and executed across multiple machines. Also, challenges in data storage and transfer over the network add to the complexity of using cloud computing systems [28].

Map-Reduce algorithms [29] using the cloud-ready framework Hadoop are available for sequence alignment and short read mapping [30], SNP identification [31], RNA expression analysis [32], amongst others demonstrating the usability of cloud services to support large-scale sequence processing. Despite emergence of methods in cloud-ready frameworks, many bioinformatics tools, analysis pipelines, and standardized methods are not readily transferable to these frameworks but are trivially parallelized using batch processing systems [8,9].

In this paper, we describe a new application, Cloud Virtual Resource (CloVR), that relies on two enabling technologies, virtual machines (VMs) and compute clouds, to provide improved access to bioinformatics workflows and distributed computing resources. CloVR provides a single VM containing pre-configured and automated pipelines, suitable for easy installation on the desktop and with cloud support for increased analysis throughput.

In building the CloVR VM, we have addressed the following technical challenges in using cloud computing platforms:

i) *Elasticity and ease-of-use*: clouds can be difficult to adopt and use requiring operating system configuration and monitoring; many existing tools and pipelines are not designed for dynamic environments and require re-implementation to utilize cloud-ready frameworks such as Hadoop;

ii) *Limited network bandwidth*: Internet data transfers and relatively slow peer-to-peer networking bandwidth in some cloud configurations can incur performance and scalability problems; and

iii) *Portability*: reliance on proprietary cloud features, including special storage systems can hinder pipeline portability; also, virtual machines, while portable and able to encapsulate complex software pipelines, are themselves difficult to build, configure, and maintain across cloud platforms.

The architecture of CloVR addresses these challenges by

i) simplifying use of cloud computing platforms by automatically provisioning resources during pipeline execution;

ii) using local disk for storage and avoiding reliance on network file systems;

iii) providing a portable machine image that executes on both a personal computer and multiple cloud computing platforms;

In the presented work, we evaluate four features of the CloVR architecture: portability across different local operating systems and remote cloud computing platforms, support for elastic provisioning of local and cloud resources, scalability of the architecture, use of local data storage on the cloud, and built process of the CloVR VM from base image using recipes.

## Implementation

### CloVR Architecture

**Figure 1:**
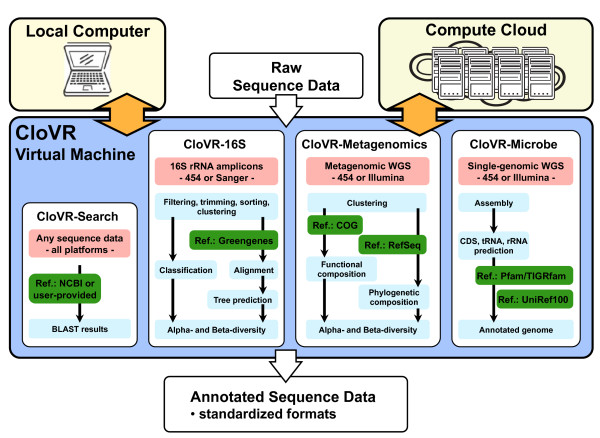
**Schematic of the automated pipelines provided in the CloVR virtual machine**. The CloVR virtual machine includes pre-packaged automated pipelines for analyzing raw sequence data on both a local computer and cloud computing platform. The primary steps in each of the four CloVR protocols are shown (light blue) along with input data (pink) and reference databases (green).

CloVR is a VM that executes on a desktop (or laptop) computer, providing the ability to run analysis pipelines on local resources (Figure 1). CloVR is invoked using one of two supported VM players, VMware [33] and VirtualBox [34]; at least one of which is freely available on all major desktop platforms: Windows, Unix/Linux, and Mac OS. On a local computer, CloVR utilizes local disk storage and compute resources, as supported by the VM player, including multi-core CPUs if available. To access data stored on the local computer, users can copy files into a "shared folder" that is accessible on both the VM and the local desktop and uses available hard drive space on the computer. Once inside the shared folder, CloVR can read this data for processing. Similarly, CloVR writes output data to this shared folder, making the pipeline output available on the desktop. This shared folder feature is supported by both VMware and VirtualBox.

**Figure 2:**
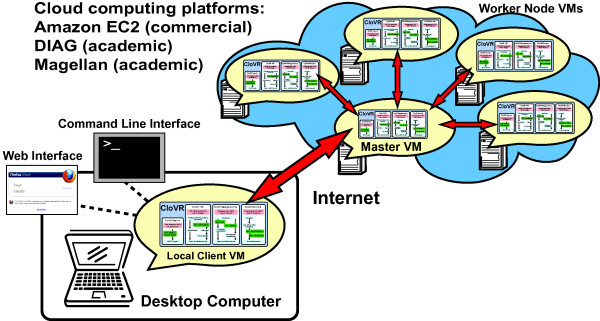
**Architecture of the CloVR application**. CloVR provides a virtual machine (VM) that is run on user's local desktop or laptop computer. The user interacts with the local VM via a command line or web interface to execute pipelines. Optionally, clusters of additional VM instances are provisioned on supported cloud platforms for increased throughput. Each cluster has a master VM instance that provides services for GridEngine [36] and Hadoop [37]. Input data and output data is transferred between the local VM and a master VM instance in the cloud over the Internet.

Optionally, the CloVR VM can be configured to automatically access a cloud provider for additional resources. Supported clouds include the commercial Amazon Elastic Compute Cloud [22] and the academic platforms DIAG [24] and Magellan [23]. In utilizing the cloud, multiple copies of the CloVR VM execute concurrently and interact as a cluster for parallel processing of data (Figure 2). Clusters of CloVR VMs running different applications on the cloud are independent and not shared between users or pipelines.

### 1.2 Components of the CloVR VM

**Figure 3:**
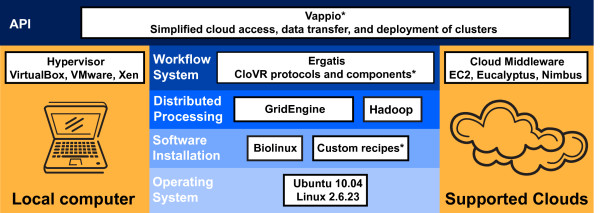
**Components of the CloVR virtual machine**. The CloVR virtual machine (blue) includes pre-installed and pre-configured software dependencies on an Ubuntu operating system to support execution on a local desktop computer and the cloud (yellow). Key software that is bundled with the VM is shown. The asterisk indicates software that was developed as part of the CloVR project.

To address technical challenges associated with software installations and pipeline configurations, the CloVR VM comes bundled with all required software pre-installed and pre-configured (Figure 3). The bundled software includes a base operating system (Ubuntu 10.04 [35] + BioLinux [3]), job schedulers (Grid Engine [36], Hadoop [37]), and a workflow system (Ergatis [9]). In addition, numerous open source bioinformatics tools are pre-installed and bundled into automated pipelines for pre-defined analysis protocols [38-40].

### 1.3 Building VM images from the CloVR skeleton VM

An automated build and configuration process is used to generate different VM images in formats compatible with both supported VM players and all supported cloud computing platforms. A specially configured VM (CloVR buildbox, http://clovr.org/developers/) running the Hudson continuous integration server [41] is used to schedule and automate the build process. Building the VM begins with a skeleton Ubuntu 10.04 disk image [42]. During the build process, a series of recipes are applied to the skeleton image to install all the necessary software, resulting in three bundles, called "base", "build" and "standard", whereby the "standard" bundle represents the CloVR disk image with all fully installed pipelines. For simplicity and maximum flexibility shells scripts are being used to build the CloVR bundles. The bundles and corresponding recipes used are in version control and freely available [43]. In order to allow external developers to modify, extend, or exchange pipelines or build custom appliances using the CloVR framework, customized recipes can be written based on the skeleton or base image. Each disk image is converted into formats for VMWare (.vmdk files) and VirtualBox (.vdi files). To speed up launching CloVR on the cloud, the raw disk image is also uploaded to Amazon EC2 (AMI format) and DIAG (Xen compatible format for cloud systems running Nimbus [44]). To support use of CloVR on Amazon EC2, our group is permanently maintaining copies of the latest CloVR image as well as several reference datasets on the Amazon Simple Storage Service (S3) [45].

### 1.4 Components of a CloVR automated pipeline

The CloVR VM (version 0.6) includes four pre-packaged and automated analysis protocols (Table 1 Figure 1), which are described in detail in the referenced SOPs: (i) a parallelized BLAST [46] search protocol (CloVR-Search version 1.0 [47]); (ii) a comparative 16S rRNA sequence analysis pipeline (CloVR-16S version 1.0 [39]); (iii) a comparative metagenomic sequence analysis pipeline (CloVR-Metagenomics version 1.0 [40]); and (iv) a single microbial genome assembly and annotation pipeline (CloVR-Microbe version 1.0 [38]). For each protocol, a limited set of configuration options and pre-defined input files are supported, such as SFF, FASTA, QUAL and FASTQ; output files are generated in standardized formats, such as FASTA and Genbank flat files, and include summary reports, tables and graphical visualizations of the analysis results.

**Table 1:** Overview of CloVR analysis protocols

Track	Process	Tool	Input	Output
**CloVR-Search**	Database search	BLAST [60]	nt or pep FASTA	BLAST output
**CloVR-Microbe **[38]	Assembly	Celera assembler [61], Velvet [51]	Raw sequence data (SFF, nt.FASTA^1^, nt.FASTQ^1^)	nt.FASTA
	Gene prediction	Glimmer3 [62]		pep.FASTA
	tRNA prediction	tRNA-scan [63]		GBK, SQN
	rRNA prediction	RNAmmer [64]		GBK, SQN
	Functional annotation	BLASTX against UniRef100 [58] and COG [65], HMMER [66] search against Pfam [67] and TIGRfam [68]		Annotated GBK, SQN
**CloVR-16S** [39]	Quality checking	Mothur [17], Qiime [18]	nt.FASTA	nt.FASTA
	Taxonomic classification	RDP classifier [69]		raw output, summary reports
	Multiple sequence alignment	Mothur, Qiime (PyNAST)		nt.FASTA alignments
	OTU clustering	Mothur (distance matrix), Qiime (uclust [70])		OTU list/table
	*α*-diversity analysis	Mothur (collectors curves, rarefaction curves, diversity and richness estimators)		summary reports/diversity curves
	*β*-diversity analysis	Metastats [71], custom R scripts, Qiime		summary reports/figures
**CloVR-Metagenomics **[40]	Clustering and artificial replicate removal	UCLUST	nt.FASTA	nt.FASTA
	Functional classification	BLASTX against COG		raw output, summary reports
	Taxonomic classification	BLASTN against RefSeq [72]		raw output, summary reports
	Comparative analysis	Metastats, custom R scripts		summary reports/figures

Abbreviations: nt, nucleotide; pep, peptide; GBK, GenBank.; SQN, Sequin (NCBI sequence submission table format);

Key bioinformatics tools utilized in each protocol are listed. For input, only the required inputs from the user for each analysis track are listed. For outputs, only the data saved from each step is listed.

^1^- Inputs may require adapter and qc trimming prior to assembly

**Figure 4:**
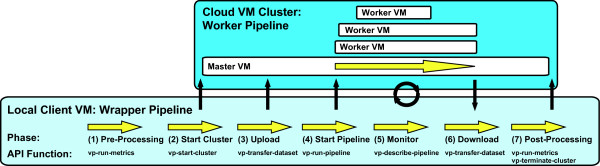
**Steps of an automated pipeline in CloVR**. A pipeline executing on the local client VM is comprised of seven primary steps. The primary API functions invoked during each step are shown with the prefix 'vp-'. For cloud-based execution, a worker pipeline is executed remotely on one or more CloVR VM instances on the cloud. The local client VM monitors the worker pipeline and VM instances on the cloud. Upon pipeline completion, output data is automatically downloaded to the local VM for viewing or post processing.

Each CloVR protocol is implemented as two discrete pipelines: i) a *worker *pipeline and ii) a *wrapper *pipeline. CloVR uses the Ergatis workflow engine [9] to describe and execute each of these pipelines. The worker pipeline implements and performs the particular analysis protocol, while the wrapper pipeline handles data management and automated use of the cloud from the desktop using the local VM client, if this execution mode is selected (Figure 4). Each wrapper pipeline is composed of seven primary phases: (1) pre-processing, including quality and integrity checks of input data; (2) starting a remote cluster for distributed processing; (3) data upload to the cloud; (4) execution of the worker pipeline locally or on the cloud; (5) monitoring of the worker pipeline; (6) data download from the cloud and (7) post-processing. Steps (2), (3), (6) are only executed when utilizing a remote cloud platform.

To implement each of these steps in the wrapper pipeline, we built a set of utilities and a web services application programming interface (API) in a software package called Vappio http://vappio.sf.net. Vappio is built on top of the Amazon EC2 API [48], which includes functions for managing images, instances, and authentication key pairs. By comparison, Vappio provides functions for managing clusters, datasets, and analysis protocols. A summary of the Vappio functions and web services follows:

#### (i) Clusters

On the cloud, clusters of CloVR VM instances are configured for parallel processing. CloVR utilizes these clusters as temporary resources during pipeline processing, provisioning a new cluster for each pipeline, and terminating the cluster upon pipeline completion. Each cluster runs an instance of both Grid Engine [36] and Hadoop [37] for job scheduling. Clusters are composed of a single master node and one or more worker nodes (Figure 2). The client CloVR VM running on the user's desktop is also considered a cluster, named 'local' that is both a master and worker type. The first VM that is started in a cluster is designated as the master node. Subsequent VMs are designated as worker nodes, automatically registered with the master node and added to the cluster upon boot of the image. Worker nodes are configured in Grid Engine queues in order to receive work units based on the number of CPUs that are available on the instance.

#### Communication between clusters

All communication and data transfer between a user's desktop and the cloud is managed by the client CloVR VM running on a local computer. The local client VM communicates with the master CloVR VM on the cloud to transfer data, invoke worker pipelines, and monitor the pipeline state (Figure 4). To provide security and help ensure data privacy, each remote cluster of CloVR VMs uses a unique, randomly generated authentication key. This key is used to enable secure data transfer between instances with Secure Shell (SSH) both within the cloud and over the Internet and between the local client VM and master cloud CloVR VMs.

#### Cluster management

To manage the cluster on the cloud, Vappio provides web services to dynamically start (*vp-add-cluster*), resize (*vp-add-instances*) and terminate (*vp-terminate-cluster*) clusters of VM instances. These web services in turn utilize EC2 API calls [48], including *ec2-run-instances*, *ec2-terminate-instances*, and *ec2-describe-instances*. In addition to executing the EC2 API calls, the Vappio web services manage the configuration of Grid Engine and Hadoop on each instance, as the instance is started and added to or terminated and removed from the cluster.

#### User authentication on the cloud

In order to access the cloud, user account and authentication information is required and obtained from the cloud provider. To simplify access to the cloud during pipeline execution and without jeopardizing security, Vappio provides a unique identifier, called a credential name, for each cloud account. During an initial configuration, the credential name is configured and associated with the cloud account and the authentication keys using the Vappio web service, *vp-add-credentials*. This credential name is then used to refer to the account in subsequent Vappio web service calls during pipeline execution.

#### (ii) Datasets

In Vappio, datasets are described as lists of files or Uniform Resource Locators (URLs) that are accessible by a cluster or the local client CloVR VM. User-provided sequence data, reference data, and output generated by the CloVR analysis pipelines are all managed as datasets. Datasets are moved between a local desktop and disk storage on the remote cluster as needed for processing (Figure 4, Steps 3 and 6). Vappio provides utilities for 1) registering new datasets with a cluster (*vp-add-dataset*), 2) transferring datasets between the cloud and local desktop (*vp-transfer-dataset*), and 3) requesting information about a dataset (*vp-describe-dataset*).

#### (iii) Protocols

**Figure 5:**
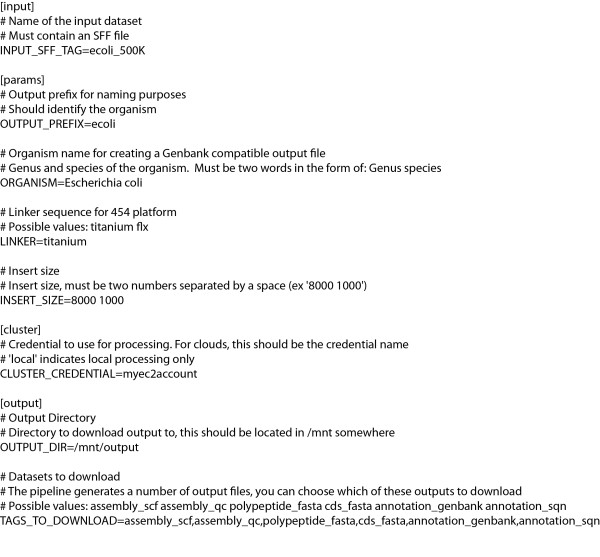
**Example of a specification file used to configure pipeline execution**.

Pre-defined analysis protocols are invoked for data analysis using a single configuration file (Figure 4, Step 4). Vappio provides utilities for configuration and invocation of analysis protocols with the services *vp-describe-protocol *and *vp-run-pipeline*. An example of the configuration file for CloVR-Microbe run on 454 sequence data produced by *vp-describe-protocol *is shown in Figure 5. The specification file includes references to input data sets, configurable analysis parameters, and, optionally, references to account credentials for accessing the cloud. Protocols are executed with *vp-run-pipeline*, which accepts the specification file as input. Once executed, the running instance of the protocol is referred to as a pipeline. The status of pipelines is monitored with the service *vp-describe-pipeline*.

#### (iv) Transparency and reproducibility

For complex pipelines, reproducibility becomes increasingly difficult and virtualization and clouds have been recognized as ideal platforms to promote pipeline reproducibility [25]. CloVR realizes this potential for reproducible research by executing all steps on a portable VM that encapsulates the entire runtime environment, included versioned protocols and analysis results.

To ensure transparency of the CloVR-supported analysis, each CloVR protocol is described by two documents: (1) An abstract workflow XML file that is used by the Ergatis workflow engine to execute the protocol and (2) a human readable standard operating procedure (SOP) document that describes the protocol in detail and has been published with open access elsewhere. The abstract workflow XML is an exact description of the executions used to perform the analysis. The SOPs describe each step of the pipeline, including software tools, software versions, and parameters used.

To ensure reproducibility of individual analysis results, CloVR uses the following additional principles: 1) All pipelines are executed using the Ergatis workflow system that tracks process flow and exact parameters invoked at each step in the XML file. 2) As part of the CloVR software installation process, versioning is applied to each analysis protocol, reference data set, and to the CloVR VM image itself. All results generated during CloVR pipeline runs have references to these versions.

### 1.5 Data storage and transfers

All input data and results generated during a CloVR pipeline execution are stored in the "shared folder" on the local client VM and can be accessed from the local computer. When using the cloud, input data is copied to the cloud as needed and output data is retrieved as part of the pipelines. To improve network transfer performance, CloVR uses high performance Secure Shell (HPN-SSH) [49] to transfer files. The synchronization utility rsync [50] is used to avoid redundant data transfers. Since all network transfers between the local desktop and the cloud are managed by CloVR VMs, data transfer is automatic, invisible to the user and does not require further software installations or configurations by the user. Upon pipeline completion, the final output is transferred from the master node on the cloud to the local VM and, subsequently, the entire cluster terminated on the cloud.

The pipelines in CloVR are configured to avoid unnecessary data transfers for both local and cloud-based execution modes. For example, publicly available reference datasets used by several of the supported protocols are either permanently hosted on the cloud or at an Internet accessible URL [38-40]. When executing CloVR pipelines on Amazon EC2, pipelines utilize reference datasets hosted on Amazon S3 where configured. For local execution, reference datasets are first downloaded to the local VM over the Internet. CloVR ensures such local transfers happen only once, the first time the data is accessed, and the reference data is then saved locally for subsequent access.

For data storage of intermediate results or temporary files during pipeline execution on the cloud, CloVR utilizes the local ephemeral disks provided to each cloud instance and does not require access to a shared file system, such as a NFS server. Under this model, worker nodes must receive copies of input data from the master node before beginning work, which is implemented using the "job prolog" feature of Grid Engine. Similarly, output data is copied back to the master node using the "job epilog" feature of Grid Engine. To provide robustness and scalability, all data transfers to and from the master node are also scheduled as jobs in Grid Engine queues named staging.q and harvesting.q. The number of slots in these queues allows for control over how many simultaneous transfers a master node will process. HPN-SSH and rsync are used to perform the transfer between instances in the cloud.

In some cases, pipelines use reference datasets or intermediate outputs that need to be accessed on every instance in a cluster. A single directory (the staging directory/mnt/staging/) is used to mirror such data to all instances in the cluster. Rather than rely exclusively on the master node to provide a mirror of the data, a custom built peer-to-peer transfer scheme is used to improve transfer throughput. Under this strategy, worker nodes share copies of the staging directory in a peer-to-peer fashion using rsync and HPN-SSH. Sun Grid Engine is used to schedule these transfers and limits the number of transfers per host, thereby avoiding overloading any single host. Upon receiving a complete copy of the staging directory, a worker nodes is added to a Grid Engine queue (named stagingsub.q) indicating that they can mirror copies to peers.

### 1.6 Automatic resource provisioning in the cloud

During execution of CloVR pipelines in the cloud, cluster sizes of CloVR VM instances are configured automatically, i.e. additional VM instances are automatically provisioned, if necessary. Pipelines that are configured to run exclusively on a local CloVR VM instance skip resource allocation steps. To determine the number of compute instances needed for the different CloVR protocols, custom scripts follow a hierarchy of the following factors: 1) hard-coded assumptions about expected resource utilizations, 2) cluster size limits set by the cloud provider, and 3) runtime estimations by the user based on input data.

An example of a hard-coded resource provisioning is the short-read Illumina sequence data assembly step using Velvet [51], which is part of the CloVR-Microbe pipeline and requires larger RAM allocations than comparable Roche/454 sequence data assemblies. When executed on the Amazon EC2 cloud, CloVR-Microbe starts a single high-memory instance type (m2.xlarge) that provides 17.1 GB of RAM, which in our testing is sufficient for assembly of single bacterial genomes. Local execution is limited by available RAM on the machine.

For three of the pre-packaged protocols in CloVR (Microbe, Metagenomics, and Search), BLAST searches are the primary processing bottleneck. In these cases, an estimation of total BLAST runtime can serve as a good approximation to predict the overall pipeline runtime. Based on our observations, BLAST runtimes can vary for a particular search database depending on the length and composition of query sequences. For the CloVR-Search and CloVR-Metagenomics protocols, total BLAST runtimes are estimated based on the input data with the Cunningham tool [52] and used to determine how many instances to start prior to search. Cunningham, which was implemented as part of the CloVR project, rapidly estimates BLAST runtime by comparing the *kmer *profiles (*k *= 3 for protein, *k *= 11 for DNA, including reverse complemented sequence) of a pre-calculated reference database and the input query sequence. First, a default minimum of five c1.xlarge instances providing a total of 40 CPUs is started to support BLAST steps in these pipelines. Second, Cunningham is used to determine the expected CPU hours required for the total BLAST search and to allocate additional machine instances, not exceeding a user configurable upper limit.

The cloud provider may impose a limit on the maximum number of instances that can be started by a user (e.g. Amazon EC2 imposes a default limit of 20 instances per account, which can be raised on request). For each CloVR pipeline, users also have the option to set an instance limit in the configuration file, which prevents attempts to start more than the specified number of instances for a particular pipeline.

Also impacting BLAST runtimes are the number and size of partitions that are used for parallel processing. In CloVR, BLAST searches are run in parallel by dividing the input query multi-FASTA files into partitions and executing a search of each partition concurrently against the reference database. Over-partitioning of the data leads to very short durations of individual jobs and can result in inefficient use of resources and increased runtimes due to the overhead in the scheduling and invocation associated with each job. Provided the Cunningham BLAST runtime estimate, the partition size *P *for each BLAST query in the CloVR pipeline is obtained by

$$P=\frac{N_{q}}{T∕R}$$

where *N_q _*is the total number of query sequences, *T *is the estimated CPU runtime from Cunningham, and *R *is a configurable parameter for the preferred execution time for a single data partition (default: 2 hours). The support for runtime estimates is provided as a configurable module that reads the pipeline configuration and produces an estimate. This allows for custom modules for runtime prediction in the future using some other logic.

## Results

### 1.7 CloVR runs on the desktop and dynamically utilizes cloud computing providers

To demonstrate the portability of CloVR, we executed a single analysis protocol (CloVR-Microbe) on a local desktop computer and two cloud computing platforms (Table 2). The input data comprised of 250,000 454 FLX Titanium 8 kb paired-end sequencing reads of the bacterium *Acinetobacter baylyi *totaling ~89 Mbp and expected to cover the ~3.5 Mbp genome at 25-fold coverage. Identical output, comprised of 38 contigs (N50: 262 Kbp) and 3,417 predicted coding genes was obtained on all three platforms. For local analysis, a CloVR instance with 4 CPUs and 8 GB of RAM was used. When using the cloud platforms, the local client VM can be executed in as little as 2 GB of RAM. The DIAG and EC2 platforms allowed for the parallelization of several steps of the protocol offering 4-CPUs per "medium" instance type on DIAG (8 GB RAM) and 8-CPUs per "c1.xlarge" instance type on EC2 (7.5 GB RAM).

**Table 2:** Portability and performance of the CloVR VM

	Local PC (Intel Xeon 5130) Max No CPUs: 4	DIAG (medium instance) Max No. instances: 5 Max. No CPUs: 20	Amazon EC2 (c1.xlarge instance) Max No. instances: 18 Max No. CPUs: 80
	Runtime	Runtime	Runtime
**Assembly**	29 min	25 min	28 min
**Annotation**	2 days 6 hrs 26 min	9 hrs 30 min	7 hrs 2 min
**Total**	2 days 7 hr 5 min	9 hrs 55 min	7 hrs 30 min

Our evaluation of the CloVR-Microbe protocol demonstrates the ability to run the same genome assembly and annotation protocol both locally and on the cloud for increased throughput (Table 2). A single configuration setting is changed to invoke the pipeline on either the local desktop or the supported clouds.

### 1.8 CloVR provides automated resource provisioning in the cloud

**Figure 6:**
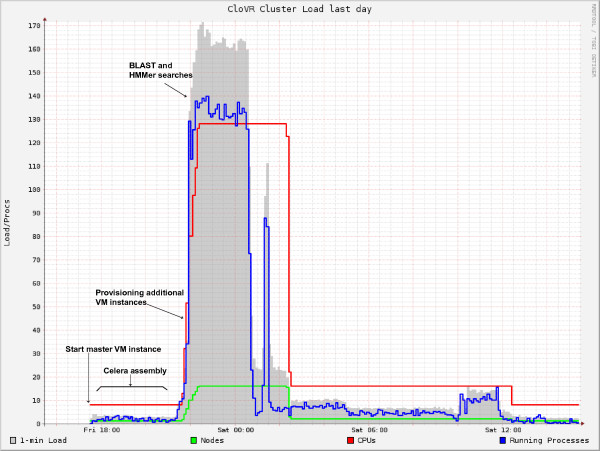
**Execution profile of an analysis with CloVR-Microbe**. CloVR-Microbe was used to perform whole-genome shotgun (WGS) assembly and annotation on 500,000 3 kbp paired-end sequence reads generated with the 454 Titanium FLX platform from a *Escherichia coli *whole-genome shotgun library (unpublished data). The local VM client first started a remote (master) VM instance on the cloud. The input sequencing reads (676 MB, compressed SFF file) were copied to this instance and assembled on a single c1.xlarge VM instance, using no more than no more than two out of the eight available CPUs. Then, prior to the genome annotation, which involves several parallelizable search steps, 15 additional CloVR VM instances were allocated to improve processing throughput. A configurable parameter limits the number of instances that are added. Idle instances are subsequently terminated automatically upon job completion on an hourly timer.

**Figure 7:**
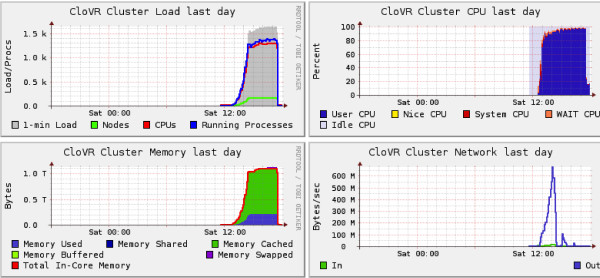
**Dynamic allocation of CloVR VM instances to a cluster on the cloud running BLAST**. A cluster of CloVR VMs is deployed on-the-fly and scaled to 160 c1.xlarge Amazon EC2 instances (totaling 1280 virtualized CPUs) running BLAST of a random sample of ~100 Million nucleotides from metagenomic whole-genome shotgun sequencing with 454 Titanium FLX of an unpublished oral microbiome project against the NCBI non-redundant protein database.

Elasticity, i.e. dynamic provisioning of resources, is a primary feature of the cloud and allows for the addition of computational resources on-demand. Figure 6 shows the automatic allocation of CloVR VM instances to the cluster on the cloud and the subsequent termination of idle instances upon job completion for the microbial genome assembly and annotation steps of the CloVR-Microbe pipeline and demonstrates dynamic capabilities provided by CloVR. Figure 7 shows a BLASTX comparison using CloVR-Search on clusters composed of up to 160 c1.xlarge instances, comprising 1,280 CPUs. This BLASTX search ran on Amazon EC2 with a throughput of ~36.9 Mbp per c1.xlarge instance, at an estimated total cost of ~$108 per hour for all 160 instances. Resource provisioning for CloVR-Microbe is automatic; for CloVR-Search it is configured by the user but does not require the direct user interaction with the remote cluster on the cloud.

### 1.9 CloVR uses local disks and does not rely on network file systems during pipeline execution

**Figure 8:**
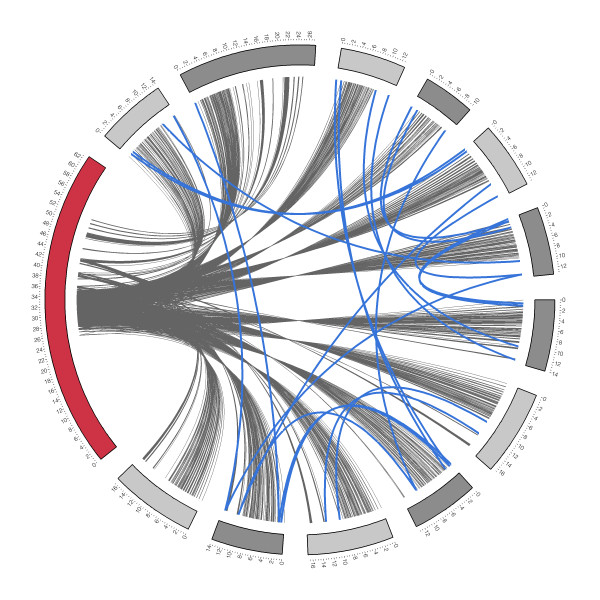
**Visualization of data transfers between instances over time in a cluster of CloVR VMs**. Each segment of the circle represents the lifetime of a single CloVR VM instance. Labels indicate time since bootup in wallclock hours. The red segment represents the master node CloVR VM and the grey segments the worker VM instances. Data transfers between master and worker instances are shown as grey lines. Transfers between worker instances are shown as blue lines.

Bottlenecks in reading or writing data on a shared, network-based file system, such as NFS [53], can cause performance problems during processing, especially when many concurrent processes are executing against the shared resource. To avoid data transfer bottlenecks CloVR uses local disks space of the instance running on the desktop or cloud, requiring input files to be transferred to each compute host within the cluster. For data input, these file transfers between master and worker node types are made prior to computation, for data output subsequent to job completion. In addition, reference data sets and intermediate outputs need to be accessed by all VM instances in a cluster. To improve distribution of these data sets, a peer-to-peer data transfer scheme is used for sharing intermediate results and reference data sets. Figure 8 shows data transfers within a cluster of 160 CloVR instances during a run of CloVR-Microbe on Amazon EC2.

**Figure 9:**
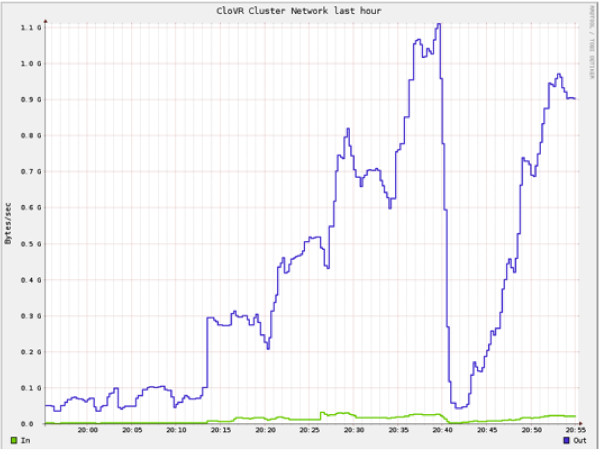
**Network throughput on a cluster of CloVR VMs on Amazon EC2**. The aggregate network throughput as measured by Ganglia [59] during a peer-to-peer data transfer on a cluster of 160 c1.xlarge instances on Amazon EC2.

To evaluate the performance of data transfers, the throughput for providing 3.1 GB of compressed reference data to a cluster of 100 c1.xlarge CloVR VM instances was tested (Figure 9). Instances came online in a staggered fashion and received copies of the reference data upon boot of the instance. The aggregate data throughput exceeded 1.1 GB per second. By comparison, network transfer speeds between a pair of c1.xlarge instance types on the Amazon EC2 network were found to typically fall below ~40 MB per second (data not shown).

## Discussion

CloVR reduces bottlenecks in sequence analysis by using two related technologies: virtual machines (VMs) and cloud computing. CloVR simplifies deployment of complex bioinformatics workflows by providing a single executable (the VM) that is portable across all major operating systems. By default, CloVR runs on a desktop but enables seamless access to large distributed computing resources including Amazon EC2 and other cloud computing platforms, providing a potential alternative to building and maintaining in-house infrastructure for computational analysis.

While genomic workbench systems focus on providing extensive customization, i.e. choices of multiple tools for easy integration into user-customized pipelines, many projects instead rely on static, standardized analysis pipelines. To accommodate this scenario, CloVR provides pre-defined standard pipelines that integrate tools for particular analysis objectives so that no additional configuration or expertise with individual tools by the user is required. This level of automation is intended to enable genomics applications for users that find choice of bioinformatics tools overwhelming and instead seek recommendations for best practices. Ongoing work includes a web-based user interface (Additional file 1), which will run locally on a user's desktop, and further simplify execution of analysis protocols.

CloVR utilizes a recipe-driven process to build VMs for both the desktop and cloud computing platforms, which allows for construction of customized VMs by other software developers. While CloVR currently includes a set of protocols for microbial genome analysis, the CloVR VM can serve as a general platform for the integration of additional tools and protocols developed by the research community. To add new protocols to CloVR, developers build recipes to install new software, deploy and test pipelines in the Ergatis workflow system, and create new configuration files for the CloVR API. A first step in this direction has been made by the use of CloVR to create a VM for the QIIME package [54]. We plan to create a wizard to simplify this process in the future and support custom repositories of build scripts.

In contrast to Internet-based software-as-a-service solutions for sequence analysis, such as Galaxy [8] or Taverna [11], which provide centralized that typically execute on dedicated resources and require users to upload data CloVR follows a decentralized model where each of multiple users executes a local client VM that is independent from other CloVR instances in a multi-user environment. By running on the local desktop, CloVR provides the opportunity to utilize substantial computing power provided by multi-core desktop CPUs, potentially avoiding the need for data transfer over the Internet and for use of the cloud entirely. The decentralized CloVR architecture saves all pipeline outputs locally on the personal computer, providing users additional controls on maintaining data privacy. Although CloVR transfers data to cloud servers for processing, CloVR uses the cloud as a temporary resource and does not require that either inputs or results are stored permanently on the cloud.

The architecture of CloVR, which utilizes Grid Engine [36] for job scheduling and local disks for storage, allows for migration of tools and pipelines from non-cloud versions to the cloud without reimplementation. This approach is in contrast to the use of tools developed for cloud-ready frameworks like Hadoop, which are algorithms that follow MapReduce [29]. The availability of these tools is, however, still relatively limited, since utilization of the Hadoop framework requires new methods or reimplementation of existing tools. As more tools that utilize MapReduce [30-32] are becoming available, Hadoop is included on the VM for their potential future integration.

CloVR provides utilities for building private clusters of VM instances on-demand in the Cloud, without expecting users start, manage, or resize clusters in the cloud. A few other systems, such as Nimbus one-click clusters [55], Galaxy CloudMan [27] and StarCluster [56], are also designed to deploy clusters of instances in the cloud. In contrast to these systems, CloVR pipelines include steps to provision these resources automatically. This enables cost savings in the case of commercial clouds, by allocating resources only as they are needed ("just-in-time"). To help ensure compatibility with multiple cloud providers and support emerging cloud computing platforms, CloVR avoids reliance on proprietary features of individual cloud providers, instead utilizing only three EC2 API calls during pipeline execution (*ec2-run-instances*, *ec2-terminate-instances*, and *ec2-describe-instances*). Such core functions of the EC2 API are becoming a standard in middleware that provides cloud services and are expected to be supported by public and private clouds.

With the increasing volume of next-generation sequencing data, data storage and transfer is increasingly important component of analysis pipelines. Compute clusters often rely on centralized, shared storage systems or file servers to simplify access to data for users and pipelines. As part of the design to be both portable and scalable on cloud computing networks, CloVR does not rely on a shared, network file system, such as NFS, for storage. Instead, CloVR relies on local disk storage on either the users' desktop to store pipeline input and output, or temporary disk storage available on the cloud VM instances during pipeline execution. By using local disk for storage rather than the network, CloVR can be expected to run on commodity cloud systems with relatively slow networking and without reliance on the specialized storage features of cloud providers, such as Amazon Elastic Block Storage [57].

Increasing data volumes can be an impediment for utilizing the cloud, as this data needs to be transferred over the Internet. A strategy for moving analysis to data, rather than transferring data over the network, has been raised as a potential solution to dealing with data transfer bottlenecks [5]. The portability of the VM provides such flexibility. The CloVR VM is 1.4 GB compressed and can be easily transferred to computational resources that are co-located with large data sets. Similarly, reference datasets can be saved on the cloud to avoid data transfers over the Internet, such as is done for Uniref100 [58], which is a 2.6 GB compressed reference dataset hosted in the cloud to support the CloVR-Microbe protocol.

The CloVR pipelines are composed of multiple steps, only some of which are computationally demanding or support parallelization on multiple CPUs. To match pipeline needs with available resources, each CloVR pipeline includes steps to automatically provision cloud resources as needed. One strategy for efficient allocation of resources is to estimate runtimes for steps that execute in parallel, in order to only provision resources that can be used. The Cunningham [52] utility, which is implemented in CloVR currently estimates BLAST runtimes during pipeline executions of CloVR-Search and CloVR-Metagenomics. This strategy helps in avoiding over-partitioning of the input data, which introduces overhead that degrades overall performance, and starting too many instances for small searches or too few instances for larger searches. The ability to predict runtimes can also be used to provide an *a priori *estimation to the user of how much an analysis will cost or whether a particular analysis is even feasible. We plan to explore providing such estimates as future work and anticipate this will be of much interest to users of the software.

## Conclusion

We have designed, built, and tested a portable virtual machine, named CloVR, that provides automated analysis pipelines for microbial genomics. CloVR provides a stand-alone client VM for execution on a personal computer providing the ability to perform sophisticated analyses using local resources and cloud computing platforms if additional resources are needed for increased throughput. By providing fully automated pipelines, the CloVR VM allows users without extensive bioinformatics background to process sequence data, lowering the barrier of entry for microbial sequence analysis.

## Availability and requirements

The CloVR VM is freely available for download from http://clovr.org

**• Project name: **CloVR

**• Project home page: **http://clovr.org

**• Operating system(s): **Platform independent

**• Other requirements: **VMWare, VirtualBox virtual machine players

**• License: **BSD

**• Any restrictions to use by non-academics: **none

## Authors' contributions

SVA and WFF conceived and designed the project with the help of OW. SVA, MM, AG implemented the supporting API and custom CloVR VM. SVA, KG, MV, DRR, CA, JRW implemented and tested the CloVR pipelines. SVA and JRW ran experiments for the paper. SVA and WFF drafted the manuscript. All authors read and approved the final manuscript.

## Supplementary Material

Additional file 1 **CloVR screencast**. A short screencast launching and CloVR and using the web interface to launch an analysis. Click here for file
